# The Role of Parental Monitoring and Affiliation with Deviant Peers in Adolescents’ Sexual Risk Taking: Toward an Interactional Model

**DOI:** 10.5812/ijhrba.8554

**Published:** 2013-06-26

**Authors:** Khodabakhsh Ahmadi, Javad Khodadadi Sangdeh, Sajad Aminimanesh, Ali Mollazamani, Mostafa Khanzade

**Affiliations:** 1 Behavioral Sciences Research Center, Baqiatallah University of Medical Sciences, Tehran, IR Iran; 2 Department of Counseling, University of Kharazmi, Tehran, IR Iran; 3 Department of Psychology, Shahid Chamran University of Ahvaz, Ahvaz, IR Iran

**Keywords:** High Risk Sex, Parenting, Peer Group, Adolescente

## Abstract

**Background:**

Adolescence is considered as an important phase for beginning sexual high risk behaviors that increases the possibility of negative, unpleasant and problematic consequences like unwanted pregnancy and probability of copulative disease transmission.

**Objectives:**

To determine the prevalence of sexual risk taking among students in Tehran and to develop and test a model for the relationship between parental monitoring and affiliation with deviant peers as they predict youth risky sexual behaviors.

**Materials and Methods:**

In this cross sectional study, 1266 adolescents were recruited from high schools in Tehran and three scales of sexual risk behavior, parental monitoring and adolescent affiliation with deviant peers were completed. Data was analyzed using independent sample t-test, Pearson correlation coefficient and structural equation modeling.

**Results:**

According to the results, about one-fifth of subjects were at high risk in terms of unsafe sexual relationships. The percent of positive attitude among males was nearly 2 times more than that of females. The investigated model for the mediating role of affiliation with deviant peers in the relationship between parental monitoring and sexual risk taking was confirmed and explained 0.32 of sexual risk taking variance.

**Conclusions:**

The results of this study suggested that parental monitoring and affiliation with deviant peers largely explained sexual risk taking among adolescents. Therefore, prevention efforts aimed at reducing risky sex should compose of these factors. In fact, the results suggested that earlier prevention efforts may be warranted.

## 1. Background

Adolescence is often described as a period of heightened reactivity to emotions paired with reduced regulatory capacities (1). Due to egocentrism and misapprehension of adolescents to their behaviors, this period is considered as an important phase for beginning high risk behaviors (2), that increases the possibility of negative, unpleasant and problematic consequences for adolescents (3). Based on previous researches, rates of various high risk behaviors such as smoking and alcohol use (4), substance abuse (5), physical aggression (6), risky driving (7), and unprotected sexual relationships (8), are increasing among adolescents. Moreover, children’s participation in risky behaviors has become one of the most important sources of concern for parents (9). Following substance and alcohol abuse, unsafe sexual relationships have the most harmful outcomes for adolescents compared to other high risk behaviors (2), which increases unwanted pregnancy rates and transmission probability of copulative diseases like AIDS (10, 11). Regarding the existing rates, it seems necessary to consider sexual risky behaviors. Levy and his colleagues suggested that 45.6% of 12 to 18 year old adolescents reported sexual contact in the previous 3 months, of whom 17.2% had Sexual contact without condoms (12). Moreover, 62.6% of American adolescents as surveyed by Baily and his colleagues’ reported engaging in sexual relationships, 11% of who were engaged in high risk sex (13). Getting older, adolescents reported higher tendency for sexual high risk relationships (14-17), for example, 33% of 13 year old girls and 75% of 15 year old girls as well as 45% of 13 year old boys and 95% of 15 years old boys were engaged in sexual relationships (17). Another study reported that 1% of 12-13 years old adolescents had been engaged in various sexual behaviors compared with 33% of 14 - 17 years old adolescents (18). Evidence shows that the mid ages of adolescence are the most critical ages to get oneself adapted with sexual high risk relationships (9). Dominant sociability theories have emphasized the role of principal resources such as family, school and peers in normal and abnormal behavior acquisition (19). Among family process variables, parental monitoring has been identified in the literature as one of the proximal determinants of early development and maintenance of antisocial and high risk behaviors in children and adolescents (20). Parental monitoring typically is defined as parent’s knowledge of the whereabouts of their teenager when they are not with them, and knowing whom they are spending time with (21). Parental monitoring can be conceptualized as parenting behaviors involving attention to and tracking of the whereabouts and doings of the adolescent (20). In researches, parental monitoring is usually operationalized as parental awareness, or adolescents’ perceptions of their parents’ knowledge, about the leisure activities and whereabouts of their offspring and friends/peer group (22). It has been well established that low levels of parental monitoring has been associated with sexual risky behaviors in adolescents (9, 23-26). Young adulthood is described as a period for increased chances of relationship with peers and entering social context and new activities (4). Achieving intimacy needs, adolescents preference to pass their time out of home with peers (27). Brendgen et al. (28), mentioned parental monitoring as an influencing factor in adolescents’ participation in risky behaviors and affiliation with deviant peers. Affiliation with deviant peers is described as the relationship with adolescents who are committing behaviors like weapon carriage, offending, and drug abuse (29). With respect to the social learning theory, relationship with deviant peers can impress adolescents’ problematic behaviors (30). Recent research shows a significant relationship between affiliation with deviant peers and sexual high risk behaviors (31-33). Poorly monitored adolescents are more likely to participate in risky behaviors (9), and may be at an ampliﬁed possibility for affiliation with deviant peers (34). Problem behavior theory and other available models on high risk behaviors propose that peer affiliation mediates the relationship between parental monitoring and adolescent problem behaviors (30). In other words, parental monitoring can impress high risk behaviors through affiliation with deviant peers (29, 35). However these studies have not considered the effectiveness of parental monitoring and affiliation with deviant peers on sexual high risk behavior in adolescents.

## 2. Objectives

To determine the prevalence of sexual risk taking among students in Tehran and to develop and test a model for the relationships among parental monitoring and affiliation with deviant peers as they predict youth sexual risk behaviors.

## 3. Materials and Methods

The sample consisted of 1266 adolescents (737 girls and 529 boys), who were recruited from high schools in Tehran, Iran. The Inclusion criteria were the following: age limitation from 14 to 18 and residency in Tehran. Participants were selected through the cluster sampling method and they completed administered questionnaires individually with regular supervision to provide reliable and valid data. The following instrumentations were applied to collect data.

### 3.1. Sexual Risk Behavior Scale

The SRBS is a 4-item self-report scale which assesses the adolescents’ attitudes to sexual relationships (36). Due to cultural limitations, there was no feasible method to assess sexual relationship record directly. Originally validated with college students, the SRBS has acceptable internal consistency (α = 0.84). In this study, the Cronbach’s α of scale was 0.67.

### 3.2. Parental Monitoring Scale (PMS)

The PMS is a seven-item self report instrument, that had previously achieved a Cronbach’s α of 0.81 (37). Parental monitoring items included questions about adolescent’s whereabouts, friends and activities. The possible responses were “never/unimportant” (0) to “always/very important” (20). For this study Cronbach’s α was 0.70.

### 3.3. Adolescent Affiliation with Deviant Peers Scale (AADPS)

The AADPS is an 8-item scale, used to ask adolescents for deviant behaviors committed by their peers, such as drug and alcohol use, carrying knife or gun and physical fighting during the past six months (29). The possible responses were “none of them (0)” to “all of them (4)”. The total response score was computed for each adolescent, with the higher score indicating more affiliation with deviant peers. The Cronbach’s α of scale was 0.82.

### 3.4. Statistical Analysis

Prevalence rates of sexual high risk behaviors were computed using descriptive analysis. Moreover, the latent variable analyses were performed using structural equation modeling which compared a proposed hypothetical model with a set of actual data. The closeness of the hypothetical model to the empirical data was evaluated statistically and is presented in Table 1.

**Table 1.** Gender Wise Comparison of Study Variables Among Students

	Males, Mean ± SD	Females, Mean ± SD	df	T-test	P Value
**SHRB**	12.16 ± 5.32	10.74 ± 4.02	936.802	-5.131	000
**PM**	21.29 ± 3.90	23.74 ± 3.08	967.141	12.049	000
**AADP**	14.58 ± 5.76	10.40 ± 3.44	794.579	-14.90	000

## 4. Results

### 4.1. Prevalence of Sexual High Risk Behavior

According to the SHRBS, about one-fifth (19.6%) of all subjects were at high risk in terms of unsafe sexual relationships. The percentage of positive attitude among males was nearly 2 times more than the prevalence among females (28.9% vs. 12.9%, chi square = 50.252, P < 0.001).

### 4.2. Sociodemographic Variables Analysis

The participants were 529 male and 737 female adolescents. The participant’s mean and standard deviation (SD) of age were 16.07 and 1.04 years for males and 16.04 and 1.22 for females, respectively. All participants were high school students and 4.5% of them reported distress in the structure of their families. The results of independent sample t-test for study variables are shown in Table 1. These findings showed that males and females were significantly different in scores of SHRB (P < 0.001), parental monitoring (P < 0.001) and affiliation with deviant peers (P < 0.001).

### 4.3. Model Testing

Table 2, shows the mean and standard deviation of the study variables and their correlations. As the table shows, there is a positive and significant relationship between SHRB and Adolescent Affiliation with Delinquent Peers (AADP) while PM in negatively correlated with SHRB and AADP.

**Table 2.** Mean and Standard Deviation of the Study’s Variables and Their Correlations (P < 0.001)

	Mean ± SD	Correlation, r^a^		
		SHRB	PM ^a^	AADP ^a^
**SHRB**	11.33 ± 4.66	1		
**PM**	22.71 ± 3.65	-.310	1	
**AADP**	12.16 ± 4.99	0.358	-0.362	1

^a^Abbreviations: PM; Parental Monitoring, AADP; Adolescent Affiliation with Delinquent Peers, r; Pearson correlation coefficient

To investigate the proposed model based on the mediating role of AADP in PM and SHRB relationship, our findings confirmed the model. Considering the obtained error index, this model explains 32% of SHRB variance. Confirming the mediating role of AADP, the model’s goodness of fit was investigated using the chi square test and Adjusted Goodness of Fit Index (AGFI). The AGFI was equaled to be 0.98. The insignificant chi-square showed model goodness of fit. Table 3, shows all of the investigated Goodness of Fit Indices.

**Table 3.** Goodness of Fit Indices of the Investigated Model

Χ^2^, df=45	Χ^2^/df	RMSEA^a^	NFI^a^	NNFI^a^	CFI^a^	RMR^a^	Standardized RMR^a^	GFI^a^	AGFI^a^
125 (P = 0.00)	2.77	0.039	0.97	0.97	0.98	0.034	0.030	0.98	0.97

^a^Abbreveations: RMSEA, Root Mean Square Error of Approximation; NFI, Normed Fit Index, NNFI, Non-Normed Fit Index; CFI, Comparative Fit Index; RMR, Root Mean Square Residual; GFI, Goodness of Fit Index; AGFI, Adjusted Goodness of Fit Index

**Figure 1.**
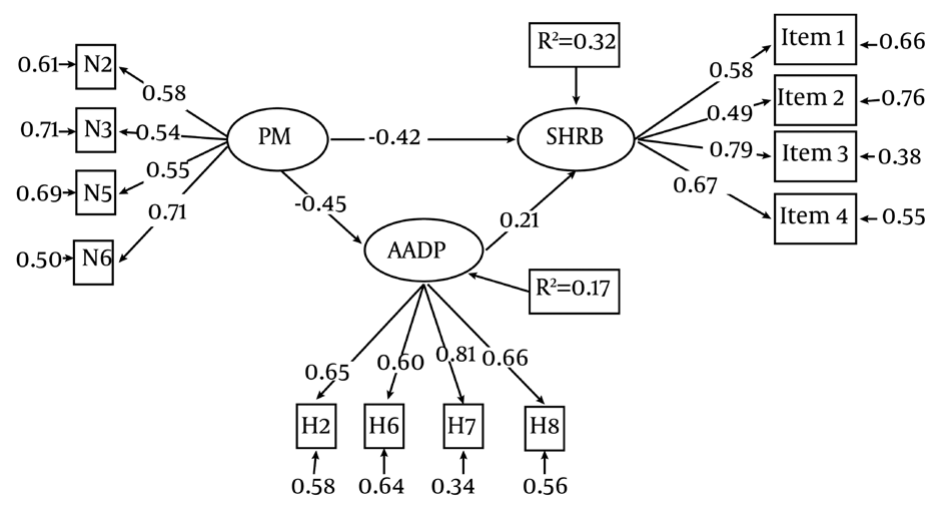
The Investigated Model for the Mediating Role of AADP in the Relationship Between PM and SHRB

Figure 1, shows the results of the investigated structural equation model. Regarding this model, PM has a significant effect on SHRB through AADP. The direct and indirect effectiveness of PM on SHRB were -0.42 and -0.094 respectively. Moreover, AADP effectiveness on SHRB was 0.21.

Schreiber et al. (38), argued that the model has goodness of fit if and only the indices of NFI, NNFI, CFI, GFI and AGFI exceed 95%, the RMR index is near zero and SRMR and RSMEA indices are smaller than 0.80% and 0.60%, respectively. Therefore, considering the claims of Schreiber et al. (38), the current model benefits from goodness of fit.

## 5. Discussion

This study aimed to investigate the prevalence of sexual risky behavior among students and the role of parental monitoring and affiliation with deviant peers in predicting sexual high risk behavior. According to the findings of this research, about one-fifth of the adolescents were at high risk, in terms of unsafe sexual relationships. This can be ascribed to factors such as psychosocial characteristics of the adolescents (39) and peers’ influence (19). Moreover, sexual activity and unprotected sex can be used as coping mechanisms in distressed teens (40). This study, being consistent with that of Rie et al. (9) and Ramer et al. (14), also showed that sexual risk taking was more common among boys compared to girls. Explaining the results, factors such as gender roles, different expectations from girls (41), and parents’ extra monitoring (20), should be taken into account. Our results, were similar to those of Brendgen, et al. (28), Paschal, Ringwalt, and Flewelling (29), and Meldrum, Young, and Werman (30), showing that affiliation with deviant peers could predict the occurrence of high risk behaviors. Consistent with previous research, spending time with deviant peers as well as its direct effect on juvenile high risk behaviors was associated with parental monitoring (28, 35). The results support the basic argument that deviant peers are an important factor in the development of juvenile high risk behaviors as suggested in the Social Learning Theory (30). In our study, we found that parental monitoring was an influential predictor of sexual high risk behaviors directly and through affiliation with deviant peers. Previous research suggested that parental monitoring is an important deterrent of sexual high risk behaviors (28, 40, 42), hence this study supported this prediction. Considering the obtained results of the current study, the theoretical model proposed by Paschal et al. (29), is confirmed. In line with previous research, it can be concluded that parental monitoring effectiveness on high risk sexual behavior is mediated through affiliation with peers (9, 29, 35). Limitations of this study are worthy of discussion. Since studies in Iran have not investigated sexual high risk behavior and its relationship with parental monitoring and affiliation with deviant peers, the obtained data from the current study cannot be compared with research carried out on Iranian samples. Moreover, taking into account cultural limitations, we investigate SHRB indirectly, which can affect the results of this study. Another limitation is that measurement of research variables was based on participants’ self-report, and there was no independent method for testing the validity of their responses. Also, this study was carried out in Tehran and its result should be generalized with caution. Future studies would probably benefit from using interview and observational research data to help researchers understand the connections of adolescent sexual high risk behavior and its connected variables in greater depth. Generally speaking, results of this study suggested that parental monitoring and affiliation with deviant peers largely explains sexual risk taking among adolescents. Therefore, prevention efforts aimed at reducing risky sex should compose of these factors. In fact, the results suggested that prevention efforts beginning earlier (i.e. at the start of high school), may be warranted.
